# Macroenzymes in Isolated Hyperenzymemia: Detection Methods and Decision Thresholds for Polyethylene Glycol-Precipitable Activity

**DOI:** 10.7759/cureus.117331

**Published:** 2026-10-01

**Authors:** Lukasz Kopoczynski, Adam Lasota, Dorota Bartkowiak, Kamil Boguszewski, Michal Wiesner, Bartlomiej Jakubczyk, Lukasz Wodzinski

**Affiliations:** 1 Medicine, Polish Red Cross Maritime Hospital, Gdynia, POL; 2 Cardiology, Pope John Paul II Regional Hospital, Zamosc, POL; 3 Medicine, Dr. Jan Biziel University Hospital No. 2, Bydgoszcz, POL; 4 Medicine, New Hospital in Naklo and Szubin, Szubin, POL; 5 Medicine, District Health Center, Malbork, POL; 6 Medicine, Ludwik Rydygier Collegium Medicum in Bydgoszcz, Nicolaus Copernicus University in Torun, Bydgoszcz, POL; 7 Medicine, Florian Ceynowa Specialist Hospital, Wejherowo, POL

**Keywords:** decision thresholds, isolated aspartate aminotransferase elevation, isolated hyperenzymemia, macroamylase, macro-aspartate aminotransferase, macro-ast, macro-creatine kinase, macroenzymes, polyethylene glycol-precipitable activity, polyethylene glycol precipitation

## Abstract

Macroenzymes, such as macroamylase, macro-aspartate aminotransferase (macro-AST), and macro-creatine kinase (macro-CK), are high-molecular-weight complexes formed when serum enzymes bind to immunoglobulins or other plasma components, or undergo self-polymerization. These complexes can artificially inflate results in routine activity assays, producing a picture of isolated hyperenzymemia, such as isolated AST elevation, and are sometimes the reason for costly, occasionally invasive diagnostic workup. A common screening method is polyethylene glycol (PEG) precipitation, where the result is reported as PEG-precipitable activity, with the percentage of activity precipitated by PEG (%PPA), and evaluated against a decision threshold.

This review examines the heterogeneity of %PPA decision thresholds and of the alternative detection methods and assesses how consistently these criteria are applied in practice. The analysis covers the literature published from March 2021 onward, supplemented by iterative backward citation searching, which brought in the earlier studies that established the %PPA thresholds, and by forward citation searching of those studies.

The thresholds compared here vary substantially for the same enzyme, with the widest discrepancies in CK. The differences stem from variations in assay platforms, reference populations, the statistical cut-offs adopted, and the precipitation protocol itself, so the thresholds are not transferable between laboratories. Within that protocol, polymer concentrations remain relatively uniform, while incubation times, centrifugation conditions, and reference materials differ substantially from report to report. Storage at 4°C, used as a lower-cost alternative, is not reliable either, since enzyme activity sometimes remains unchanged despite the presence of a macroenzyme. Reported detection rates vary by several orders of magnitude, reflecting the degree of preselection of the study group rather than the frequency of the macroenzyme itself.

A negative result from PEG precipitation or from storage at 4°C therefore does not rule out a macroenzyme. Because a %PPA value depends on the protocol, the platform, and the control sample assayed in parallel, standardized reporting of these variables is needed before the results of macroamylase, macro-AST, and macro-CK can be compared between laboratories.

## Introduction and background

The 1964 study by Wilding et al. is considered the first report on macroenzymes [1]. The authors described a female patient in whom persistently high serum amylase activity was due to the enzyme’s binding to globulin, rather than to a disease of the pancreas, kidneys, or salivary glands [1]. The higher molecular weight of macroamylase compared with free amylase impedes its glomerular filtration, thereby prolonging its half-life in the circulation; as a result, serum enzyme activity may remain persistently elevated in the absence of organ damage [2,3]. Routine measurement of enzyme activity does not make it possible to distinguish between macroenzyme activity and free enzyme activity. Macro-aspartate aminotransferase (macro-AST), that is, a complex of AST with immunoglobulin, is listed among the extrahepatic causes of increased aminotransferase activity in the American College of Gastroenterology guidelines table [4]. Aside from this mention, the guidelines do not specify when to consider a macroenzyme, which test to order, or how to interpret its result.

Macroenzymes are classified into type 1 and type 2 [3,5]. Type 1 is a complex of an enzyme with immunoglobulin class G (IgG), A (IgA), or M (IgM), and the mechanism by which it forms remains unclear [5,6]. Molecular mimicry is one of the proposed models: an antibody raised against an exogenous antigen cross-reacts with a structurally similar endogenous enzyme and binds it through the Fab fragment [5,6]. The model comes from a case in which macro-AST appeared after the start of specific allergen injection immunotherapy, a treatment that raises allergen-specific IgG; the macroform accounted for approximately 95% of total AST activity in a patient whose AST had previously been normal, although the authors presented the link as speculation [6]. The site of the interaction has been localized experimentally: papain digestion of purified anti-enzyme antibodies yields Fab fragments that retain their enzyme-binding capacity [2], an observation later confirmed independently by Moriyama’s team using the same method [7]. The mass of the complexes, assessed by gel filtration and ultracentrifugation, corresponds to two enzyme molecules per immunoglobulin molecule, the ratio expected for a specific immune interaction [2]. Macro-AST has a molecular weight of approximately 250 kDa [5]. Type 2 covers the remaining forms, in which the enzyme is associated with non-immunoglobulin components or exists as an oligomeric complex of an isoenzyme [3].

Elevated enzyme activity is not a prerequisite for the presence of a macroenzyme. In a review by Remaley and Wilding, elevated amylase activity was found in only two of 16 patients with macroamylase, and elevated total creatine kinase (CK) activity was found in fewer than 0.5% of those with macro-CK [2]. More recent data confirm this: in the study by Bürki et al., a sample in which gel filtration chromatography (GFC) detected macro-CK had an initial CK activity of 83 U/L, which fell within the reference range, with a percentage of activity precipitated by polyethylene glycol (PEG; %PPA) of 83.1% [8]. Šimac et al., in a series of patients with rheumatoid arthritis, described cases in which increased aminotransferase activity resulting from a macroenzyme was initially diagnosed as methotrexate hepatotoxicity, and treatment was modified [9]. However, since a macroenzyme can coexist with activity within the reference range, its presence does not automatically explain every subsequent change in test results: the authors recommend that any subsequent acute or rapid change in enzyme activity be evaluated independently, as it may reflect a real pathology superimposed on the known macroenzyme [9].

The boundary between the physiological form of the enzyme and the macroform is not always clear-cut. Bürki et al. noted that gamma-glutamyl transferase (GGT) circulates physiologically in four forms differing in molecular weight, three of them lipoprotein-associated: big-GGT (above 2,000 kDa), medium-GGT (940 kDa), small-GGT (140 kDa), and free GGT (70 kDa), with the free fraction being virtually absent [8]. Each of the four samples tested had a different distribution of GGT among these forms, which hampered the identification of a sample containing macro-GGT. For this enzyme, the boundary between the physiological form and the macroform therefore remains unclear [8].

No clear causal relationship has been demonstrated between the macroenzyme and a specific disease. Remaley and Wilding summarized the state of knowledge by stating that there is no convincing evidence of the pathogenicity of anti-enzyme antibodies [2]; Galasso et al. regarded the associations of macro-CK with malignancy and macro-lactate dehydrogenase (LDH) with autoimmune disease as hypotheses requiring prospective confirmation [10]. This assessment remains valid, and more recent studies also identify type 1 macroenzymes as having diagnostic, but not clinical, significance [8].

Precipitation with PEG is routinely available in laboratories, primarily due to its use in the determination of macroprolactin, which supports the expansion of this method to include enzymes [5]. Macrocomplexes have also been described for other non-enzymatic analytes: an antibody bound to the analyte alters its availability to the reagent and distorts the assay result, as has been documented for high-sensitivity cardiac troponin T and in insulin autoimmune syndrome [11,12].

A %PPA result depends on two things: the protocol by which the precipitation is carried out and the decision threshold against which the value is read. Reported thresholds differ between enzymes, analytical platforms, and reference populations, so the same sample may be called positive in one laboratory and negative in another. This review compares the protocols behind the reported values, maps the decision thresholds in circulation, sets the alternative detection methods alongside PEG precipitation, and examines how consistently these criteria are applied in the reports that use them.

## Review

Literature selection methodology

This paper is a narrative review that compares the %PPA decision thresholds determined independently in four datasets with the universal conventions in use in the literature and with screening methods that serve as alternatives to PEG precipitation.

Reports were eligible for inclusion if they concerned macroenzymes in humans, presented primary data, were published in English, Polish, or French, and reported at least one of the following as a value not already represented in this review: a %PPA decision threshold, a parameter value of the PEG precipitation protocol, a detection rate in a defined population, or a series of enzyme activities measured after storage at 4°C. The value contributed by each included report is given in Table 1. Whether a value was already represented was judged against the values compiled in the comparison tables and discussed in the text, taken as a whole, rather than against the order in which reports were screened. Reports concerning macrocomplexes of non-enzymatic analytes fell outside this scope and are cited as background only.

**Table 1:** Reports meeting the inclusion criteria. Inclusion criterion met: T - a %PPA decision threshold; P - a value of a PEG precipitation protocol parameter; D - a detection rate in a defined population; S - a series of enzyme activities measured after storage at 4°C. Codes are given in the order T, P, D, and S. In the last column, table and figure numbers indicate where the report is tabulated or plotted, and text indicates that it is discussed in the body of the review without appearing in any comparison table. Reports are listed in order of citation. AST - aspartate aminotransferase; ALT - alanine aminotransferase; CK - creatine kinase; PEG - polyethylene glycol; %PPA - percentage of enzyme activity precipitated by PEG

Report	Design and study group	Enzyme(s)	Inclusion criterion met	Value contributed	Use in this review
Aleknavičiūtė-Valienė et al. 2023 [5]	Case report	AST	P	Lowest centrifugal force in the protocol comparison, 554 × g	Table 3
Bürki et al. 2023 [8]	Reference-range study, 211 samples	Eight enzymes	T, P	99th-percentile thresholds for eight enzymes; the ≥80% rule	Tables 3, 4
Šimac et al. 2021 [9]	Case series, rheumatoid arthritis	ALT, AST	P, D	Detection rate in rheumatoid arthritis	Tables 2, 3
Nadour et al. 2023 [13]	Retrospective review, 42,540 patients	AST	T, P, D	Lowest detection rate; threshold above 70%	Tables 2, 3
Patteet et al. 2012 [14]	Case report and method evaluation	AST	T, P, D	Threshold as a 95% confidence interval; two detection rates	Tables 2, 3
Lee et al. 1994 [15]	Consecutive CK isoenzyme electrophoresis requests	CK	D	Detection rates for macro-CK, type 1 and type 2, in consecutive electrophoresis requests	Table 2
Moriyama et al. 1990 [16]	Patients with AST/ALT ratio of >2.0	AST	D	Detection rate at an AST/ALT ratio above 2.0	Table 2
Magen-Rimon et al. 2022 [17]	Children with chronic isolated AST elevation	AST	D	Only detection rate reported with a borderline fraction	Table 2
Čásenská et al. 2023 [18]	Case series and detection methods	Amylase	P, D, S	Only macroamylase detection rate and storage series	Tables 2, 3, 5
Caropreso et al. 2009 [19]	Children with isolated AST elevation of >6 months	AST	T, P, D	Two thresholds from one dataset, 73.3% and 75.0%	Tables 2, 3
Marczak-Karpina et al. 2020 [20]	Unexplained hyperenzymemia, 122 patients	Six enzymes	P, D	Detection rate across several macroenzymes	Tables 2, 3
Woynarowski et al. 2024 [21]	Case series, cystic fibrosis	ALT, AST	P, D	Highest detection rate	Tables 2, 3; Figure 4
Wyness et al. 2011 [22]	Reference intervals, six enzymes	Six enzymes	T	Only thresholds obtained with PEG 8000	Table 4
Davidson and Watson 2003 [23]	Method development	Seven enzymes	T, P, S	Thresholds for seven enzymes; first storage series	Tables 3, 4, 5
Levitt and Ellis 1982 [24]	Method development, macroamylase	Amylase	T, P	Origin of the 73% and 52% convention	Table 3
Dedeene et al. 2023 [25]	Upper reference limits, eight enzymes	Eight enzymes	T, P	97.5th-percentile thresholds; second-highest centrifugal force	Tables 3, 4
Litin et al. 1987 [26]	Case series, three patients	AST	P	Only protocol reporting centrifugation as high speed	Table 3
Li et al. 2022 [27]	Case report	AST	P	Longest incubation; fourfold dilution correction	Table 3
Pei et al. 2023 [28]	Case report, three replicates	AST	P, S	Only storage series performed in replicate	Tables 3, 5
González Raya et al. 2019 [29]	Case report and storage criterion	AST	P, S	The storage criterion; control range 27.6-48.8%	Tables 3, 5
Woynarowski et al. 2023 [30]	Case report, cystic fibrosis	ALT, AST	P	The limits applied with this protocol, 74% and 55%, differing from the source cited	Table 3
Sharma et al. 2024 [31]	Case report	Lipase	P	Macrolipasemia; the only protocol centrifuged at 4°C	Table 3
Čásenská et al. 2025 [32]	Critical limits, 126 sera	Enzymes and proteins	T, P	Age-dependent critical limits; the 60% convention	Table 3
Xu and Han 2025 [33]	Case report	AST	P	Highest rotational speed, 15,000 rpm	Table 3
Heo et al. 2026 [34]	Case report with a comparator patient	AST	P, S	Storage series with a macroenzyme-negative comparator	Tables 3, 5
Zhang and Han 2026 [35]	Two case reports	AST	P, S	Discordant storage results under one protocol	Tables 3, 5
Wyness et al. 2011 [36]	Method comparison, macro-CK and macroamylase	CK, amylase	T	Macro-CK and macroamylase thresholds in the PEG 8000 series; agreement with electrophoresis and ultrafiltration	Tables 4, 6
Chtioui et al. 2010 [37]	Case report	AST	S	First storage series showing no decrease	Table 5
Beşer et al. 2014 [38]	Case series, eight children	AST	S	Largest storage series in children; storage as the only method	Table 5
Yu et al. 2014 [39]	Case series, three adults	AST	P, S	Same samples precipitated at two PEG concentrations	Tables 3, 5
Ono et al. 2019 [40]	Case report with controls	AST	S	Largest control group in the storage comparison	Table 5
Fermon et al. 2025 [41]	Case report and titration experiment	AST	P	Pyridoxal phosphate titration; polymer other than PEG 6000	text
Davidson and Scott 2012 [42]	Service audit, 255 referred samples	CK	T, P, D	Proposed revision of the laboratory's own CK threshold; detection rate among referred samples	Tables 2, 3; Table 4 legend
Saracoglu et al. 2021 [43]	Case report, chemotherapy	Lipase	P	Highest centrifugal force, 13,000 × g	Table 3

The PubMed database was searched on 16 March 2026 using the term “macroenzymes,” with the results limited to the preceding five years. The search was repeated on 26 August 2026 before submission, with the publication date window set explicitly to 16 March 2021 through 26 August 2026, so that the set of records would not shift with the relative five-year filter. The initial search returned 24 records, and the repeated search returned 26, the two additional records having been published in the interval.

Records were screened in three stages. Titles and abstracts were first assessed against the eligibility conditions relating to species, data type, and language. Eligibility against the content criteria was then assessed on the abstract and, where the abstract did not state whether a threshold, a protocol parameter, a detection rate, or storage data were reported, on the full text. Reports that reported such a value were then compared with the values reported in the other retrieved reports and were retained only where at least one of their values was not among them. Of the 26 records, 14 met the inclusion criteria, 10 were excluded, and two concerned macrocomplexes of non-enzymatic analytes and are cited as background.

As a check on the sensitivity of the search term, the search was also run with a broader set of terms (“macroenzyme,” “macroenzymes,” “macro-AST,” “macroamylase,” “macroamylasemia,” and “macro-CK”) over the same period. This returned 212 records, of which 26 duplicated the primary search. The remaining 186 were screened at title and abstract level. A large proportion concerned postoperative hyperamylasemia after pancreatic surgery or endoscopic retrograde cholangiopancreatography and were outside the scope of this review. Two reports contributed a value not already represented and were included.

Backward citation searching was performed iteratively on the reference lists of the reports identified in the database searches and of the reports identified from them, to a depth of three citation steps. Reference lists were scanned by title, and publications whose titles indicated a macroenzyme topic were retrieved and assessed in full. In this way, 24 publications were assessed, of which 15 met the inclusion criteria, and nine were cited as background. This route is the source of the earlier original studies establishing %PPA thresholds that predate the search window, namely two of the four datasets compared in this review.

Forward citation searching was performed on the four datasets that established the decision thresholds compared in this review. Citation lists were taken from Europe PMC and supplemented by a web search for citing reports not indexed there. The search retrieved 69 unique citing records, of which 22 were already cited in this review. Of the remaining 47, 14 concerned analytes outside the enzyme scope, 29 reported no value of a kind listed in the inclusion criteria, and four were assessed in full text. One further citing report, not indexed in Europe PMC, was identified by the web search and assessed as well. Of these five reports, two met the inclusion criteria and were included, two reported no value that was not already represented, and one was published in Spanish and therefore did not meet the language criterion.

The Polish Medical Bibliography (Polska Bibliografia Lekarska, Main Medical Library, Warsaw, Poland) was searched separately using the term “makroenzymy” to cover Polish-language journals that are not indexed in PubMed. The search returned one record, which met the inclusion criteria and was included.

Where a report could be retrieved from more than one source, it was attributed to the source through which it was first identified. In total, 34 reports met the inclusion criteria. A further 11 publications are cited without meeting the inclusion criteria: clinical guidelines and review articles, which do not present primary data; reports on macrocomplexes of non-enzymatic analytes, which fall outside the scope of the review; and reports that describe no protocol parameter value, threshold, detection rate, or storage series. The flow of report selection is summarized in Figure 1.

**Figure 1:**
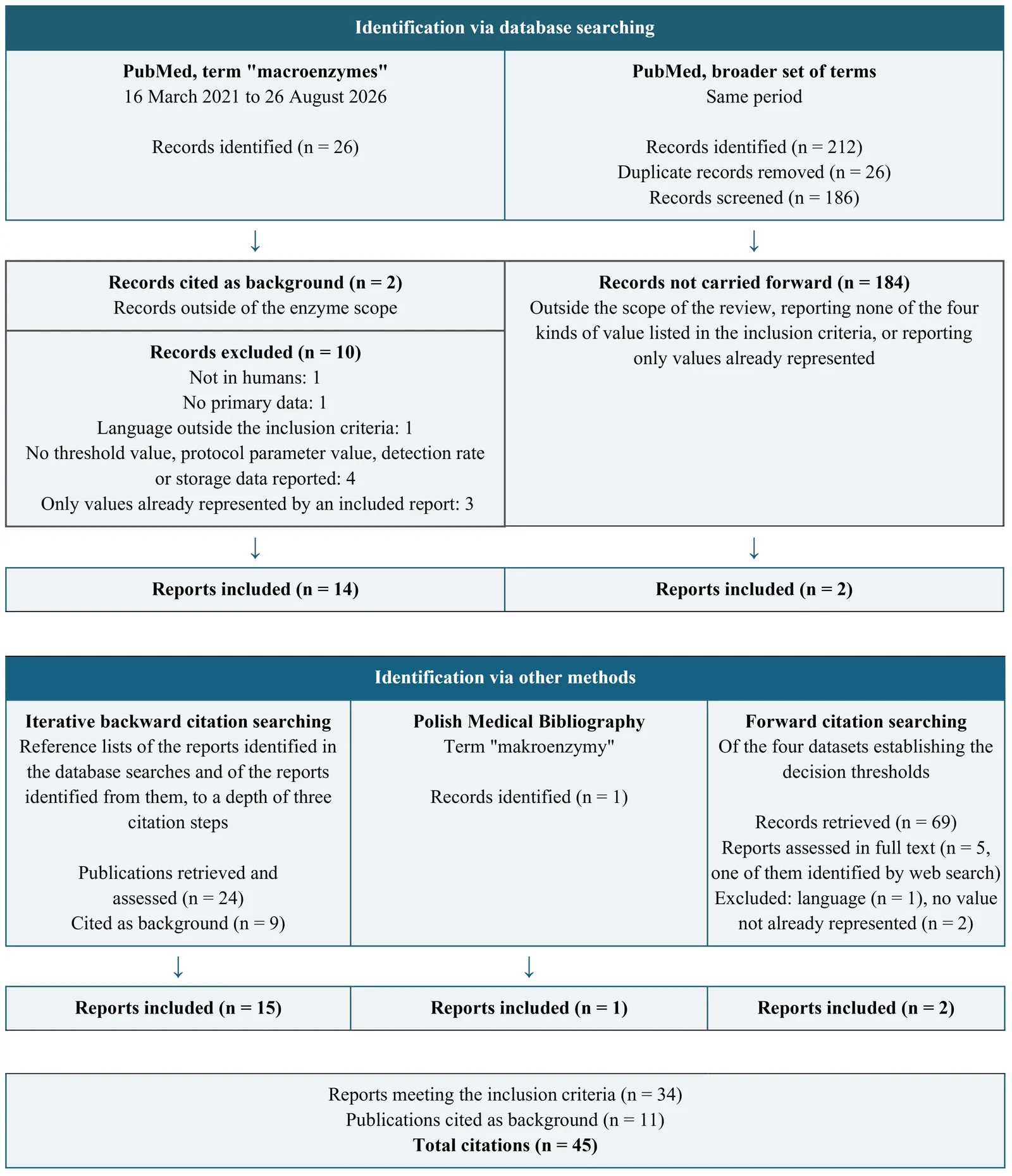
Flow of report selection. The diagram summarizes how reports were identified and selected. It is presented for transparency and does not represent a systematic review protocol; the review is narrative, and the criteria applied at each stage are stated in the Literature selection methodology section. n - number of records or reports

All 34 reports meeting the inclusion criteria are listed in Table 1, together with the criterion each of them satisfied, the value it contributes, and the place in which it is used in this review.

Detection rate and population selection

The raw data values reported in the literature are summarized in Table 2 and Figure 2.

**Table 2:** Frequency of macroenzyme detection as a function of the degree of preselection in the study group. “Positive/tested” is the number of positive results relative to the number of samples tested. Percentages are reported according to the original data; the values are not directly comparable because they differ in terms of diagnostic method, threshold, and population selection. (a) Values calculated from the source study data; the authors noted that their material did not allow an estimate of frequency and that the value would be an underestimate. PEG - polyethylene glycol; GFC - gel filtration chromatography; RA - rheumatoid arthritis; CFTR - cystic fibrosis transmembrane conductance regulator; AST - aspartate aminotransferase; ALT - alanine aminotransferase; CK - creatine kinase

Population	Macroenzyme	Positive/tested	Percentage	Method	Report
Unselected patients	Macro-AST	1/42,540	0.0024%	PEG precipitation	Nadour et al. 2023 [13]
Unselected patients	Macro-AST	1/7,273	0.014% (a)	PEG precipitation	Patteet et al. 2012 [14]
Patients with CK isoenzyme electrophoresis ordered	Macro-CK type 1	36/8,322	0.43%	Electrophoresis	Lee et al. 1994 [15]
Patients with CK isoenzyme electrophoresis ordered	Macro-CK type 2	100/8,322	1.20%	Electrophoresis	Lee et al. 1994 [15]
Patients with long-standing isolated AST elevation	Macro-AST	1/11	9.09% (a)	PEG precipitation	Patteet et al. 2012 [14]
Samples referred to a macroenzyme detection service	Macro-CK (28 type 1, two type 2)	30/255	11.8%	Isoenzyme electrophoresis (%PPA in parallel)	Davidson and Scott 2012 [42]
Patients with an AST/ALT ratio of >2.0	Macro-AST	34/260	13.1%	Electrophoresis + high-performance liquid chromatography	Moriyama et al. 1990 [16]
Children with chronic isolated AST elevation	Macro-AST	7/32	21.9% (+43.8% borderline results)	PEG precipitation + ultrafiltration	Magen-Rimon et al. 2022 [17]
Adults with suspected macroamylasemia	Macroamylase	5/13	38.5%	PEG precipitation + GFC	Čásenská et al. 2023 [18]
Children with isolated AST elevation persisting for >6 months	Macro-AST	17/44	38.6%	Electrophoresis	Caropreso et al. 2009 [19]
Patients with unexplained hyperenzymemia	Various macroenzymes	53/122	43.4%	PEG precipitation	Marczak-Karpina et al. 2020 [20]
Patients with RA and elevated aminotransferases	Macro-ALT	5/11	45.5%	PEG precipitation	Šimac et al. 2021 [9]
Cystic fibrosis patients with an ALT/AST “flare” while on CFTR modulators	Macro-ALT and macro-AST	3/3	100%	PEG precipitation + electrophoresis	Woynarowski et al. 2024 [21]

**Figure 2:**
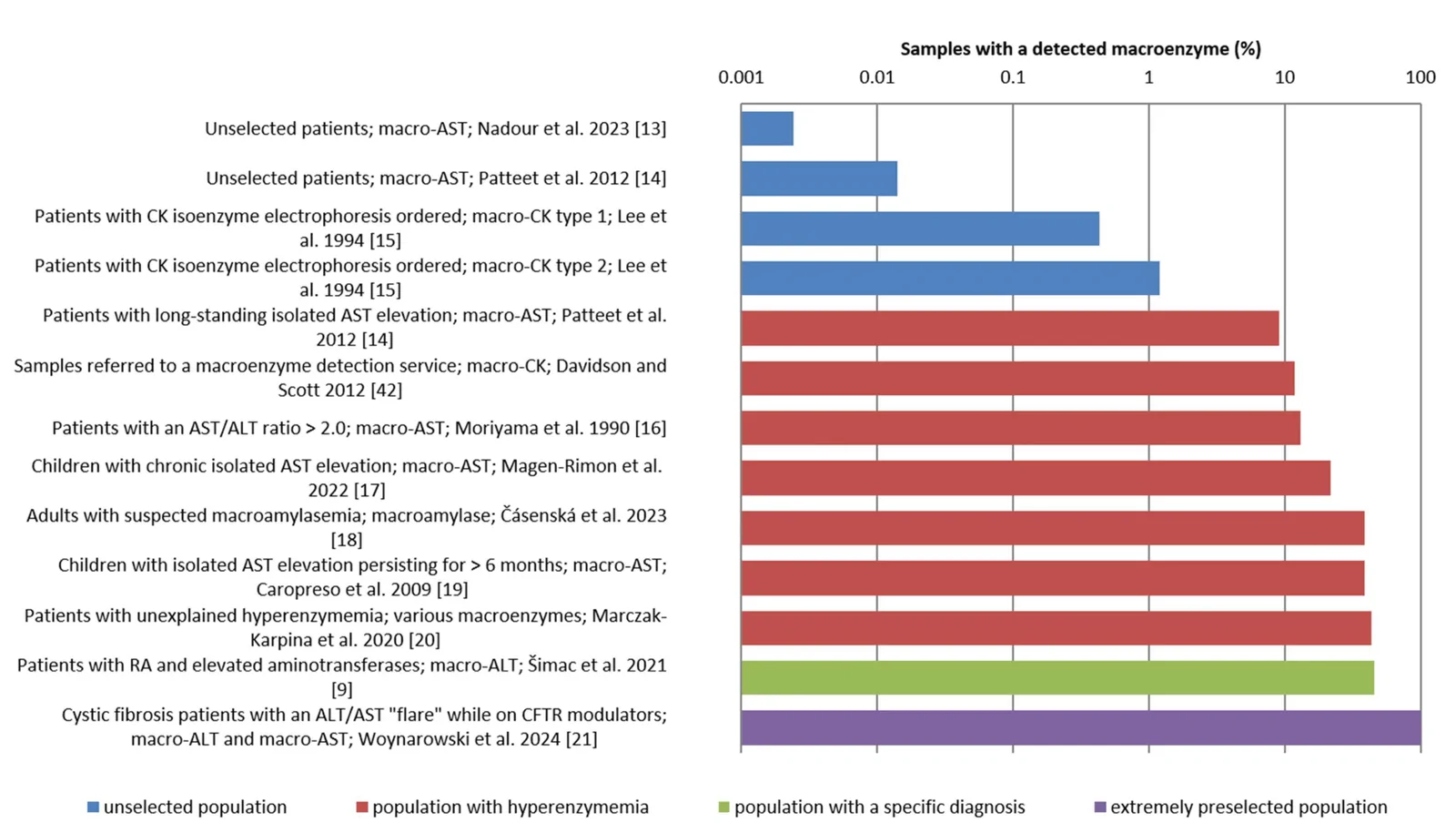
Detection rate of macroenzymes as a function of the study population (logarithmic scale). Prepared by the authors based on data from Table 2. The colors indicate the degree of preselection of the study group: blue - unselected population; red - population with hyperenzymemia; green - population with a specific diagnosis; purple - extremely preselected population. CFTR - cystic fibrosis transmembrane conductance regulator; RA - rheumatoid arthritis; AST - aspartate aminotransferase; ALT - alanine aminotransferase; CK - creatine kinase

The range of more than four orders of magnitude is influenced by the method used to select the study subjects. The lowest values come from laboratory data that were not selected based on suspicion of a macroenzyme: in the study by Nadour et al., which involved a retrospective review of all adult patients [13], and in the study by Lee et al. on consecutive requests for CK isoenzyme electrophoresis [15]. Limiting the group to individuals with hyperenzymemia not explained by organ pathology is associated with a rate several orders of magnitude higher [17,19], and the highest values come from groups defined by a specific clinical event: elevated aminotransferases in patients with rheumatoid arthritis [9] or an increase in their activity during therapy with cystic fibrosis transmembrane conductance regulator (CFTR) modulators [21]. In the last of these groups, the macroenzyme was confirmed in all subjects, which reflects the inclusion criterion adopted.

The diagnostic criteria varied across the different studies: electrophoretic separation [19], consistent results from PEG precipitation and ultrafiltration [17], PEG precipitation with reference ranges according to Wyness et al. [20,22] or with the Davidson and Watson thresholds [9,23]. Two values calculated from the study by Patteet et al. warrant separate comment [14]. The authors examined 11 samples selected from 7,273 AST assays using the PEG precipitation method and obtained one positive result, explicitly noting that their data did not allow the calculation of a frequency and that the obtained value would be an underestimate. For this reason, the values of 0.014% and 9.09%, corresponding to the ratios 1/7,273 and 1/11 calculated from these data, should be treated as an illustration of the order of magnitude, not as a frequency [14].

PEG precipitation: discrepancies in methodology and interpretation

PEG is a polymer that binds water, which acts as a solvent for plasma proteins. Reducing the amount of available water lowers the solubility of proteins, and this effect increases with their molecular weight [14,24]. The macroenzyme, as a complex with a mass many times greater than that of the free enzyme, loses its solubility at a lower polymer concentration than the free enzyme and precipitates first. The activity of the sample containing the macroenzyme decreases to a greater extent compared with the sample containing the free enzyme. This phenomenon is called volume exclusion. The difference in activity measured before and after the addition of the polymer is a measure of the proportion of macromolecular forms and forms the basis of the result, expressed as %PPA [23,25]: $\%PPA = \frac{\text{activity in a sample diluted with 0.9\% NaCl} - \text{activity in the supernatant after PEG treatment}}{\text{activity in a sample diluted with 0.9\% NaCl}} \times 100$

Extreme values are easy to interpret: a %PPA close to 0% means that adding the polymer did not reduce the activity, and thus all activity comes from the free enzyme (the presence of a macroenzyme is unlikely); a %PPA close to 100% means that virtually no activity remained after precipitation, i.e., the enzyme was completely precipitated. The difficulty in interpretation concerns the intermediate values.

The protocols used in the analyzed reports are summarized in Table 3. The PEG concentration is relatively constant at 24-25%, which corresponds to 12-12.5% after mixing with serum. This convention is not recent: Levitt and Ellis established it in 1982, precipitating amylase in a final PEG concentration of 12% obtained by mixing serum with a 24% solution of PEG 6000 in a 1:1 ratio, after preliminary experiments in which polymer concentration and incubation temperature were varied [24]. Litin et al. used the same concentration five years later [26]. The mixing ratio is 1:1 in all studies listed in Table 3 where it is specified. This concentration appears to represent a compromise: a higher concentration would also precipitate some of the free enzyme and inflate the result, while a lower concentration might fail to precipitate the complex. The remaining parameters vary widely and are given in Table 3. Centrifugation ranges from 554 to 13,000 × g, or from 3,000 to 15,000 rpm, and from 5 to 30 minutes. Incubation ranges from none reported to 20 minutes at 37°C, and the reference material ranges from saline through deionized or distilled water to phosphate-buffered saline or an undiluted sample, with or without a correction factor.

**Table 3:** Polyethylene glycol precipitation protocols in studies conducted using PEG 6000. This summary includes reports in which PEG 6000 was used, or the type of polymer was not specified. Reports stating a polymer of a different molecular weight were not included because the different molecular weight makes the remaining protocol parameters incomparable. Reports are listed chronologically. Thresholds are reported as stated in the cited studies; in the source study by Davidson and Watson, the upper limits are 76% for ALT and 53% for AST [23]. (a) In the source paper, the centrifugation time was reported in two places; the value from the Methods section was used in this summary. The reports by Woynarowski et al. [21,30] come from the same group; the 2023 report describes a single patient and states the protocol in full, while the 2024 report describes three patients and repeats only the polymer concentration, the formula, and the cut-off values; therefore, the remaining protocol parameters shown in its row are taken from the 2023 report. PEG - polyethylene glycol; URL - upper reference limit; GFC - gel filtration chromatography; AST - aspartate aminotransferase; ALT - alanine aminotransferase; NaCl - sodium chloride; rpm - revolutions per minute; × g - multiple of Earth’s gravitational acceleration

Report	PEG (type, concentration, ratio)	Incubation	Centrifugation	Reference material	Threshold used (source)
Levitt and Ellis 1982 [24]	PEG 6000, 24% in distilled water, 1:1	37 °C, 10 min	5,000 × g, 10 min, room temperature	Distilled water 1:1	At least 73% in macroamylasemic sera, less than 52% without (own work)
Litin et al. 1987 [26]	PEG (type not specified), 24%, 1:1	37°C, 10 min	High speed (not quantified), 20 min, room temperature	Water 1:1	No numerical threshold; all activity reported as precipitated
Davidson and Watson 2003 [23]	PEG 6000, 240 g/L (24%), 1:1	Room temperature, 10 min	not specified	Plasma + 9 g/L (0.9%) NaCl 1:1	Enzyme-specific range (own work)
Caropreso et al. 2009 [19]	PEG 6000, 24%, ratio not specified	Not specified	Not specified	Not specified	73.3% from the sensitivity-specificity crossing point; 75.0% from a logistic model (own work)
Patteet et al. 2012 [14]	PEG 6000, 250 g/L (25%), 1:1	Vortexing 1 min	3,000 rpm, 30 min	Serum + phosphate-buffered saline 1:1	95% confidence interval for %PPA 14.5-62.5% (own work)
Davidson and Scott 2012 [42]	PEG 6000, 240 g/L (24%), 1:1	Room temperature, 10 min	4,500 rpm, 10 min	Plasma or serum + 9 g/L (0.9%) NaCl 1:1	Positivity defined by isoenzyme electrophoresis; 45% proposed as a screening cut-off (own work, revised from 37%)
Yu et al. 2014 [39]	PEG (type not specified), 24% or 20% m/v, ratio not specified	Not specified	5,000 × g	Saline	No numerical threshold
González Raya et al. 2019 [29]	PEG 6000, 250 g/L (25%), 1:1 (procedure of Patteet et al. modified)	Vortexing 1 min	3,000 rpm, 10 min	Serum + phosphate-buffered saline 1:1	73% attributed to other authors, not derived here; control samples ranged 27.6-48.8%
Marczak-Karpina et al. 2020 [20]	PEG 6000, 24%, ratio not specified	Not specified	Not specified	Serum diluted with distilled water	Ranges according to Wyness et al. [22]
Šimac et al. 2021 [9]	PEG 6000, 25%, 1:1	37°C, 10 min	3,000 rpm, 20 min, room temperature	Deionized water	76% (ALT)/54% (AST) according to Davidson and Watson [23]
Saracoglu et al. 2021 [43]	PEG 6000, 25%, 1:1	Room temperature, 10 min	13,000 × g, 5 min	Undiluted sample, no dilution correction	Recovery below 32%, that is %PPA above 68%, according to Wyness et al. [22]
Li et al. 2022 [27]	PEG 6000, 24%, 1:1	37°C, 20 min	3,000 rpm, 5 min	Supernatant diluted 1:1 with 0.9% NaCl (correction × 4); control patient sample in parallel	%PPA ≥ 75% according to Caropreso et al. [19]
Dedeene et al. 2023 [25]	PEG 6000, 25%, 1:1	37°C, 10 min	10,900 × g, 5 min (a)	Serum + 0.9% NaCl 1:1	enzyme-specific URL (own work)
Bürki et al. 2023 [8]	PEG 6000, 250 g/L (25%), 1:1	Vortexing 1 min	9,600 × g, 10 min	Undiluted sample (correction × 2)	99th percentile (own work); ≥80% rule
Čásenská et al. 2023 [18]	PEG 6000, 25%, 1:1	Not specified	10,000 × g, 10 min	Serum + NaCl solution 1:1 and a control sample	73% according to Levitt and Ellis [24]
Aleknavičiūtė-Valienė et al. 2023 [5]	PEG (type not specified), 25%, 1:1	Vortexing 1 min	554 × g, 10 min	Serum + 0.9% NaCl 1:1	PEG as a screening test without a numerical threshold; GFC indicated as the confirmatory method
Nadour et al. 2023 [13]	PEG 6000, 240 g/L (24%), 1:1	Not specified	Not specified	Sample diluted 1:2; seven control samples in parallel	>70% (source not stated)
Pei et al. 2023 [28]	PEG 6000, 25%, 1:1	Vortexing 1 min, then 10 min (temperature not specified)	3,000 rpm, 10 min	Serum + 0.9% NaCl 1:1 (with 6 control samples in parallel)	>73% positive; <48% negative [29]
Woynarowski et al. 2023 [30]	PEG 6000, 25%, 1:1	37°C, 10 min	3,000 rpm, 20 min, room temperature	Deionized water	74% (ALT)/55% (AST) according to Davidson and Watson [23]
Sharma LK et al. 2024 [31]	PEG (type not specified), 25% in phosphate-buffered saline, 1:1	Room temperature, 10 min	3,000 rpm, 30 min at 4 °C	Serum + distilled water 1:1	No numerical threshold; recovery of 30.6% interpreted as positive
Woynarowski et al. 2024 [21]	PEG 6000, 25%, 1:1	37°C, 10 min	3,000 rpm, 20 min, room temperature	Deionized water	74% (ALT)/55% (AST) according to Davidson and Watson [23], or 61% (ALT) according to Dedeene et al. [25]
Čásenská et al. 2025 [32]	PEG 6000, 1:1 (procedure according to Levitt and Ellis [24]; concentration not specified)	Room temperature, 10 min	10,000 × g, 10 min	Serum + saline 1:1; plus a matched control sample	Assessment of the commonly used value of 60%; enzyme- and age-specific critical limits (own work)
Xu and Han 2025 [33]	PEG 6000, 25%, 1:1	Vortexing 1 min, then 10 min equilibration	15,000 rpm, 25 min	Undiluted sample (correction × 2); a control sample assayed in parallel	73.3% according to Caropreso et al. [19]
Heo et al. 2026 [34]	PEG 6000, 240 g/L (24%), 1:1	Room temperature, 10 min	3,000 × g, 10 min, room temperature	Plasma + 9 g/L saline 1:1	>73% according to Caropreso et al. [19]
Zhang and Han 2026 [35]	PEG 6000, 25%, 1:1	Vortexing, then 10 min (temperature not specified)	10,000 rpm, 10 min	Serum + saline 1:1	>73% positive, <48% negative, according to González Raya et al. [29]

Dedeene et al. identified the test used, along with calibrator traceability, protocol variability, and the type of PEG used, as the reasons why the reference ranges are not transferable between laboratories [25]. The type of polymer is determined by the number in its name, which corresponds to the average molecular weight in daltons. Table 3 focuses on studies using PEG 6000, while studies based on PEG 8000, such as that by Wyness et al., were excluded from the compilation [22,36]. They are retained in the threshold comparison in Table 4 because the point of that comparison is precisely that different protocols yield different thresholds. The strength of the volume exclusion effect is determined by both concentration and chain length; therefore, a polymer with a different molecular weight at the same percentage concentration yields a different %PPA for the same sample [18]. Yu et al. precipitated the same samples at two polymer concentrations: %PPA was 93.8%, 97.8%, and 89.7% at 24% PEG against 85.5%, 89.1%, and 84.5% at 20% PEG in the same three patients, and fell from 36.4-48% to 22.4-24% in the control samples [39]. Bürki et al. recommend that laboratories using an analyzer other than the one in their study determine their own cut-off values [8].

**Table 4:** %PPA decision thresholds proposed in four independent original studies. The upper limits of the %PPA ranges are given; these were determined in each study using its reference samples, and the values were rounded to whole numbers. The age-dependent critical limits published later by Čásenská et al. on a fifth analytical platform are discussed in the text rather than tabulated here [32]. The laboratory of Davidson and Scott later recalculated their CK limit: the robust method of the International Federation of Clinical Chemistry gave 47%, and receiver operating characteristic analysis gave 46%; the authors proposed 45% as the working cut-off [42]. (a) Hitachi 717/Modular (Roche Diagnostics, Mannheim, Germany); n ≈ 40 per enzyme; mean plus two standard deviations of the %PPA values (analyzer named by Bürki et al.; not stated in the original study) [8]. (b) Cobas 8000 c702 (Roche Diagnostics, Basel, Switzerland); n = 39-55; 97.5th percentile. (c) Alinity c (Abbott Laboratories, Abbott Park, IL, USA); n = 51-137 assays per enzyme (211 samples total); 99th percentile. The range of sample sizes is taken from the table in the source study. (d) Modular Analytics P (Roche Diagnostics, Indianapolis, IN, USA); values were converted from the recovery percentage (%PPA = 100 - recovery); the threshold was taken as the complement of the lower limit of the 95% reference interval for recovery in apparently healthy subjects; precipitation was performed using PEG 8000 in phosphate-buffered saline rather than PEG 6000; AST, ALT, ALP, GGT, LDH, and lipase from [22], CK and amylase from [36]; the metric is the inverse of that used in the other studies. (e) Total amylase. (f) Pancreatic amylase. PEG - polyethylene glycol; %PPA - percentage of enzyme activity precipitated by PEG; AST - aspartate aminotransferase; ALT - alanine aminotransferase; ALP - alkaline phosphatase; CK - creatine kinase; LDH - lactate dehydrogenase; GGT - gamma-glutamyl transferase

Enzyme	Davidson and Watson 2003 [23] (a)	Dedeene et al. 2023 [25] (b)	Bürki et al. 2023 [8] (c)	Wyness et al. 2011 [22,36] (d)	Range (percentage points)
AST	53%	48%	61%	74%	26
ALT	76%	61%	70%	92%	31
ALP	36%	29%	17%	50%	33
CK	37%	24%	36%	64%	40
LDH	70%	65%	45%	82%	37
GGT	51%	55%	41%	50%	14
Amylase	60% (e)	26% (e)	45% (f)	62% (e)	36
Lipase	Not investigated	56%	56%	68%	12

Two case reports from the same department, published within one year, describe different protocol parameters: centrifugation at 10,000 rpm for 10 minutes and at 15,000 rpm for 25 minutes, a saline-diluted control and the undiluted sample with a correction factor, two formulations of %PPA, and thresholds attributed to two different sources [33,35]. Variation of this kind is therefore not confined to comparisons between centers.

Partial precipitation of the free enzyme always occurs, which limits the specificity of the method: the median %PPA values in the study group of Bürki et al. (samples with elevated activities, mostly without macroenzyme) reach 38.3% for alanine aminotransferase (ALT) and 30.5% for lipase [8]. The consequence of this phenomenon can be seen in the data of Davidson and Watson [23]. Of the 16 patients classified as negative for macro-CK, three had %PPA values above the upper limit of the CK reference range proposed by these authors, which is 37% (values of 41.0, 41.4, and 44.0). In two of these patients, electrophoretic separation did not detect macro-CK, making them false-positive results; in the third patient, electrophoresis was not performed. For comparison, the value indicated by the authors as the next in order, 66.1%, came from a sample with electrophoretically confirmed type 2 macro-CK [23].

A false-positive %PPA result may be caused by an excess of immunoglobulins in the serum, which can cause the free enzyme to precipitate along with them [25]. Čásenská et al., using their standard protocol (25% PEG 6000, 1:1 ratio), measured %PPA for total serum immunoglobulins rather than for the enzyme-bound fraction and obtained 88.6% for IgG, 80.2% for IgM, and 58.0% for IgA. These values were similar in all 13 suspected samples and in the controls, which were sera from other patients with similar amylase activity but without macroamylase. Samples with and without macroenzyme are distinguished only by the %PPA of the enzyme itself, which in this study averaged 89.1% for amylase in samples with macroamylase compared with 29.6% in the controls [18].

%PPA decision thresholds

Based on four independent datasets, specific thresholds were determined, that is, thresholds defined separately for each enzyme and analyzer, differing in both analytical platform and cut-off statistics: Davidson and Watson used the mean plus two standard deviations of %PPA (for alkaline phosphatase (ALP) after square root transformation, for CK after logarithmic transformation), Dedeene et al. used the 97.5th percentile, and Bürki et al. used the 99th percentile of the %PPA distribution in reference samples [8,23,25]. The fourth source consists of the work by Wyness et al., in which %PPA is not determined, but rather the recovery rate, that is, the activity remaining in the supernatant [22]. Wyness et al. provide nonparametric reference intervals for recovery in apparently healthy subjects (without a diagnosed disease, but also without verification); therefore, their values were converted to %PPA as the complement of the lower limit of the interval, according to the relationship %PPA = 100 - recovery [36]. The values converted in this way are the highest among the four sets for all enzymes except GGT [22,36]. A comparison of the same four datasets, using the same conversion of the data of Wyness et al. from recovery to %PPA, was later published by Čásenská et al., who added critical limits of their own from a fifth analytical platform, ranging from 23.7% for ALP to 70.4% for ALT [32]. The compilation presented here was assembled independently from the primary sources. Table 4 additionally gives the between-study range for each enzyme and includes the ALP threshold reported by Dedeene et al. and the pancreatic amylase threshold reported by Bürki et al. [8,25].

In addition to enzyme-specific thresholds, universal conventions are used in the literature. The oldest of these is the 73% threshold, sometimes used as a universal threshold for all macroenzymes, derived from Levitt and Ellis’s work on macroamylase, in which all sera containing macroamylase precipitated at least 73% of the activity, while sera without it precipitated less than 52% [24]. The threshold for AST was determined in a study by Caropreso et al.: in a group of 44 children with chronic isolated AST elevation, a threshold of 73.3% yielded a sensitivity of 88.2% and a specificity of 88.9%, with an area under the receiver operating characteristic curve of 98.1%, and the zone of equivocal results covered %PPA from 67.1% to 82.2% [19]. That report gives two values from the same dataset: 73.3% from the projection of the sensitivity-specificity crossing point and 75.0% as the optimal threshold derived from a logistic model, and later reports cite one or the other. A comparable divergence affects the lower bound. González Raya et al. quote the 73% cut-off from other authors and report %PPA in their control samples ranging from 27.6% to 48.8%; in subsequent reports, the upper end of that control range appears as a boundary below which macro-AST is considered excluded [28,29,35]. The numerical similarity between 73% and 73.3% is coincidental, as the first value was derived for amylase and the second for AST in children. The similarity between these two values does not confirm the validity of using the 73% threshold as a universal threshold for detecting other macroenzymes in other populations. In addition to the 73% threshold, two other universal rules exist: Bürki et al. propose treating a %PPA of at least 80% as indicating a very high likelihood of a macroenzyme for any given enzyme [8], while Čásenská et al. describe a %PPA above 60% as making a serum macrocomplex very likely for total and pancreatic amylase, AST, ALT, GGT, lipase, and LDH, and identify CK and ALP as the only exceptions, with critical limits set well below 60% [32]. The values are summarized in Table 4 and shown in Figure 3.

**Figure 3:**
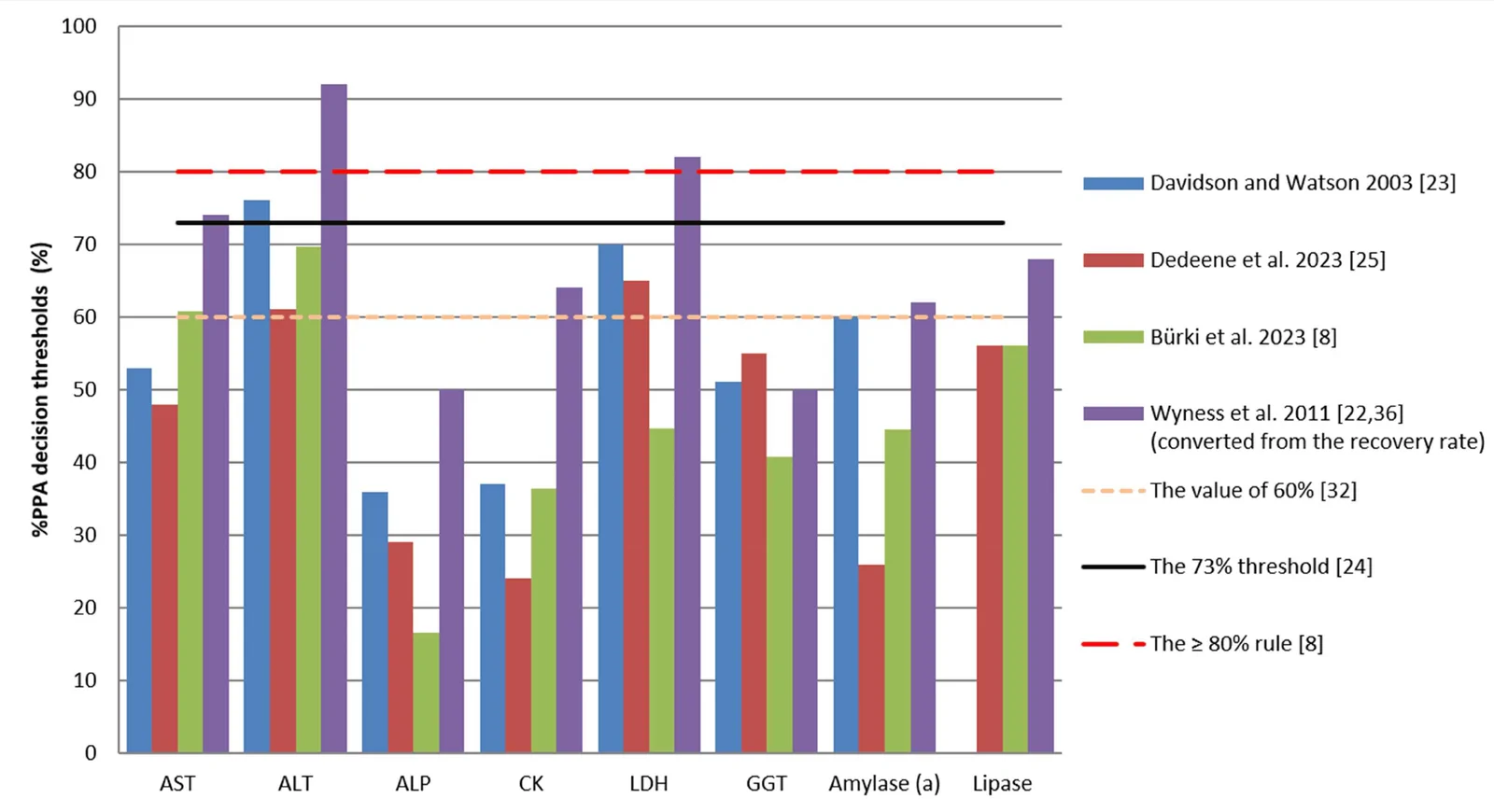
%PPA decision thresholds for eight enzymes according to four independent studies. Davidson and Watson [23], Dedeene et al. [25], and Bürki et al. [8] report values directly as %PPA; the values of Wyness et al. [22,36] were converted from the recovery rate. The horizontal lines indicate the universal thresholds used in the literature: the 73% threshold derived from Levitt and Ellis’s work on macroamylase [24], the value of 60% assessed by Čásenská et al. [32], and the ≥80% rule of Bürki et al. [8]. The limit for CK from Davidson and Watson was later proposed to be raised by the same laboratory from 37% to 45% [42]. (a) Total amylase (Davidson and Watson, Dedeene et al., and Wyness et al.) versus pancreatic amylase (Bürki et al.). Prepared by the authors based on the values compiled in Table 4. %PPA - percentage of activity precipitated by polyethylene glycol; AST - aspartate aminotransferase; ALT - alanine aminotransferase; ALP - alkaline phosphatase; CK - creatine kinase; LDH - lactate dehydrogenase; GGT - gamma-glutamyl transferase

The range of thresholds between the four datasets [8,22,23,25,36] extends from 12 percentage points for lipase to 40 for CK (Table 4). When the converted values of Wyness et al. are excluded, the range narrows to between 0 percentage points for lipase and 34 for amylase; the systematically higher thresholds obtained with PEG 8000 are therefore themselves an indication of the influence of the polymer used. None of these studies can be considered methodologically inferior to the others. Thus, four conventions coexist in the literature: thresholds specific to the enzyme and the analyzer [8,22,23,25,36], the 73% threshold from macroamylase studies [24], a 60% threshold with exceptions for certain enzymes [32], and the “≥80%” rule [8]. Thresholds also drift within a single laboratory: nine years after proposing the range for CK, the same laboratory recalculated it on 225 macroenzyme-negative samples and proposed raising the upper limit from 37% to 45% [42].

The limited clinical data support the use of specific thresholds. Dedeene et al. tested their own thresholds on three archived samples with macroenzymes confirmed by electrophoresis: %PPA was 69% and 43% in two samples with macro-CK and 52% in a sample with macro-ALP, all of which are above the enzyme-specific thresholds (24% for CK and 29% for ALP), whereas with a threshold of 73% and the “≥80%” rule, all three would be negative [25].

Woynarowski et al. described three patients with cystic fibrosis in whom an increase in aminotransferase activity occurred during CFTR modulator therapy, initially interpreted as hepatotoxicity. In all three patients, the presence of macro-ALT and macro-AST was subsequently detected by PEG precipitation and electrophoresis; the %PPA for macro-ALT was 92%, 70%, and 64%, and the %PPA for macro-AST was 75%, 61%, and 67% [21]. Figures 4A, 4B show a comparison of these values with the thresholds.

**Figure 4:**
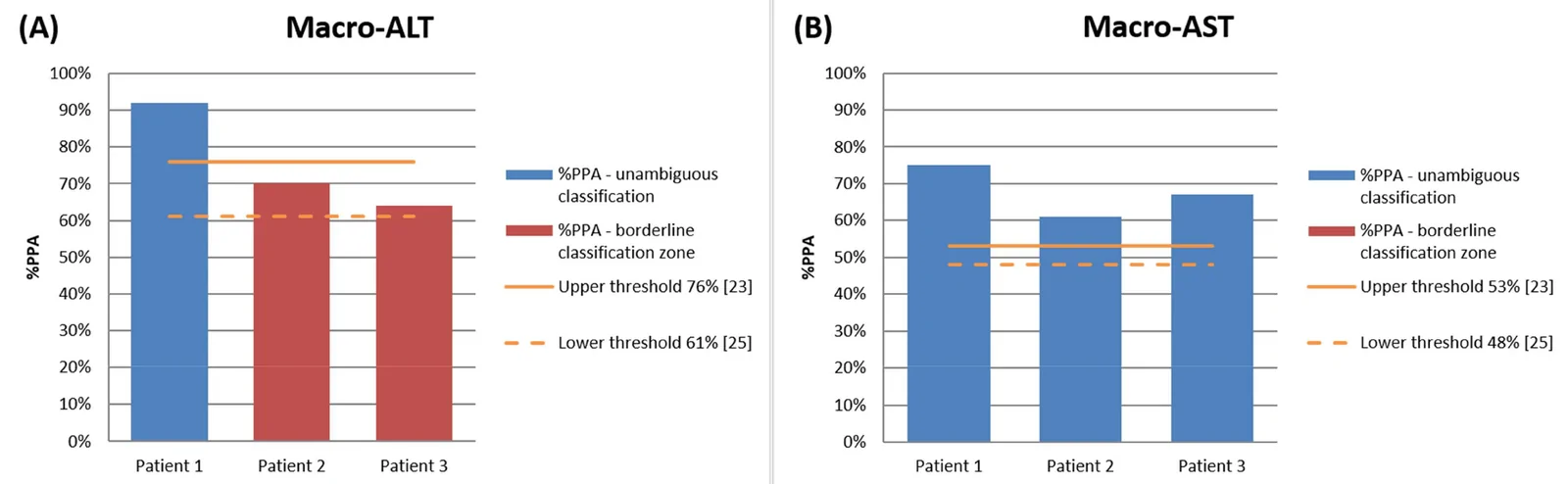
The effect of the choice of %PPA decision threshold on the diagnosis of macro-ALT and macro-AST in three patients with cystic fibrosis. (A) Macro-ALT. (B) Macro-AST. Prepared by the authors: %PPA values for the three patients from Woynarowski et al. [21]; threshold lines from Dedeene et al. and Davidson and Watson [23,25]. %PPA - percentage of activity precipitated by polyethylene glycol; ALT - alanine aminotransferase; AST - aspartate aminotransferase

Using the Davidson and Watson threshold for ALT (76%), one of the three patients would be macro-ALT-positive, while the other two would test negative despite electrophoretic confirmation; using the Dedeene et al. threshold (61%), all three would be positive [23,25]. In this situation, a negative result would mean maintaining the diagnosis of hepatotoxicity and discontinuing the CFTR modulator despite the presence of the macroenzyme. The authors report their results against both thresholds without indicating a preference [21].

Storing the sample at 4°C

The second screening method involves storing the sample at 4°C. This method is based on the lower stability of the complex compared with the free enzyme. González Raya et al. proposed a decrease in activity of at least 40% after 24 hours or 65% after 48 hours as a criterion [29]. The 10 series in which samples were stored at 4°C are summarized in Table 5.

**Table 5:** Results of the method involving sample storage at 4°C in subsequent reports. Values considered by González Raya et al. to be highly suggestive of macro-AST [29]: a decrease of at least 65% after 48 hours or at least 40% after 24 hours. The authors note that the method has not been validated, and a negative result must be confirmed by another method. In the series by Beşer et al. [38], the samples were stored at 2-8°C, and storage was the diagnostic method, applied after liver, cardiac, and muscle disease had been ruled out. A pathological control means a control test performed on a sample with elevated enzyme activity but without suspicion of a macroenzyme. A healthy control means a control test on a sample with activity within the reference range. An independent method in the column “Independent method of demonstrating the macroenzyme” means any method other than storage at 4°C, including screening methods. %PPA - percentage of activity precipitated by polyethylene glycol; PEG - polyethylene glycol; AST - aspartate aminotransferase; GFC - gel filtration chromatography; IgG - immunoglobulin G; U/L - units per liter

Report	Enzyme	Study participants	Time at 4°C	Change in activity in the test sample	Control	Independent method of demonstrating the macroenzyme
Davidson and Watson 2003 [23]	AST	One adult woman	6 days	Decrease of >90%	One pathological control (AST 174 U/L), no decrease	AST isoenzyme electrophoresis
Chtioui et al. 2010 [37]	AST	One adult woman	7 days	No significant decrease	One pathological control and one healthy control, no decrease	Adsorption on protein A Sepharose
Beşer et al. 2014 [38]	AST	Eight children	5 days	Decrease of >50% in 8 of 8	Eight controls matched for activity, decrease < 5%	No independent method, diagnosis based on storage
Yu et al. 2014 [39]	AST	Three adults	24 hours	Decrease of 92%, 56%, and 93%	Four controls, no decrease	PEG precipitation (%PPA 89.7-97.8%) and a two-year follow-up
González Raya et al. 2019 [29]	AST	One adult woman	7 days	Decrease of 46% after 24 hours, 72% after 48 hours, and 88.1% after 7 days	Five controls, decrease < 0.72% after 7 days	PEG precipitation (%PPA 98.1%), GFC at an external laboratory was negative; in the same sample, 5 days after collection, AST activity was 37 U/L compared with 494 U/L measured at baseline
Ono et al. 2019 [40]	AST	One adult woman	3 days	Decrease of 79.5%	16 pathological controls, decrease 0.61 ± 1.23%; one healthy control, decrease 3.2%	Immunoglobulin (IgG) absorption and immunofixation electrophoresis
Čásenská et al. 2023 [18]	Amylase	Five adults	5-8 days	No decrease	Controls matched by amylase activity, no decrease	PEG precipitation (%PPA, >73%) and GFC
Pei et al. 2023 [28]	AST	One adult man (three samples)	7 days	Change of 0.21-0.32%, with an increase in activity in one sample	Six controls, no decrease	PEG precipitation (%PPA, 84.6%)
Heo et al. 2026 [34]	AST	One adult woman (a second patient without macro-AST tested in parallel)	7 days	Decrease of 76% (310 to 74 U/L)	Four controls, no decrease; the second patient, without macro-AST, showed a decrease of 5-9%	PEG precipitation (%PPA, 100%)
Zhang and Han 2026 [35]	AST	Two adults	7 days	Decrease of 38.9% after 24 hours, 54% after 48 hours, and 78.7% after 7 days in the first patient; no decrease in the second	Two controls, no decrease	PEG precipitation (%PPA, 95.2% and 85.7%)

The results show a bipolar distribution. In seven series, the decrease in activity was pronounced and ranged from 50% to over 90%, while activity in the control samples remained virtually unchanged [23,29,34,35,38-40]. The largest control group was used by Ono et al., in whose study 16 samples from patients with diagnosed liver, heart, and biliary tract diseases showed an average decrease of 0.61 ± 1.23%, compared with 79.5% in the test sample [40]. In three of the 10 series, no decrease was found at all despite a confirmed macroenzyme, and in a fourth series, one of two patients with a positive %PPA showed no decrease [18,28,35,37]. Pei et al. repeated the assay three times on newly collected serum samples from the same patient, obtaining a change in activity of the order of 0.21-0.32%, including a slight increase in one of the replicates [28], which argues against an error of a single measurement. In all three studies with negative results, the heterogeneity of the complex was cited as an explanation: the class of bound immunoglobulin and the physicochemical properties of the specific complex [18,28,37]. The reports summarized in Table 5 do not describe any false-positive results; however, the control groups consisted of between one and 17 samples, which makes it impossible to estimate the specificity. The clearest illustration comes from Zhang and Han, who tested two patients with %PPA values of 95.2% and 85.7% under the same protocol: activity in the first fell by 78.7% over seven days, while activity in the second remained unchanged [35]. The method does not require reagents and can be performed even in primary care [40], but its diagnostic value is limited to positive results [18,37].

Confirmatory methods

Among the methods for detecting macroenzymes, PEG precipitation and ultrafiltration serve as screening tests, while GFC and immunofixation electrophoresis serve as confirmatory methods [5,18,36]. Wyness et al. compared both screening methods with electrophoresis: the agreement of PEG precipitation with electrophoresis in detecting macro-CK was 79.8% and that of ultrafiltration was 80.9%, with the latter exhibiting lower imprecision and narrower reference ranges; for macroamylase, the same number of positive samples was detected by both methods, but PEG precipitation yielded more equivocal results [36]. For this reason, a positive PEG precipitation result is not equivalent to confirmation of the presence of the macroenzyme and requires a reference method [36]. Storage at 4°C, described above, belongs to the first of these groups and is compared alongside them. GFC also allows typing of the immunoglobulin class: Čásenská et al. used this method to confirm the presence of IgA-bound macroamylase in three patients and IgG-bound macroamylase in two; no IgM-bound complex was detected in this group of five patients [18]. Čásenská et al. and Aleknavičiūtė-Valienė et al. propose that PEG precipitation be made available locally as a first-line test, with confirmatory methods centralized in reference laboratories serving the region [5,18]. The performance characteristics of all these methods are summarized in Table 6.

**Table 6:** Methods for detecting macroenzymes: a comparison of performance characteristics. “Time” column: “short” means a result within a single work shift; “medium” means up to one business day; and “long” means more than one business day or the need to ship the sample. The cost refers to equipment and reagents other than the routine biochemical analyzer. Sources for individual rows are listed in the “Practical notes” column. PEG - polyethylene glycol; CK - creatine kinase; GFC - gel filtration chromatography; kDa - kilodalton

Method	Principle	Time	Cost	Practical notes
PEG precipitation	Precipitation of enzyme-immunoglobulin complexes by volume exclusion	Short	Low	First-line screening test; 79.8% agreement with electrophoresis for macro-CK [36]; lack of harmonization of thresholds among laboratories [8,25]
Storage at 4°C	Lower stability of the complex than of the free enzyme	Long	Very low	Requires no reagents [40]; diagnostic value is limited to positive results [18,28,29,37]
Ultrafiltration (100 kDa filter)	Separation by molecular weight	Medium	Medium	Less imprecision and narrower reference ranges than PEG precipitation [36]
GFC	Separation by molecular weight with activity detection in the fractions	Long	High	Reference method; allows typing of the immunoglobulin class; requires a reference laboratory [5,8,18]
Immunofixation electrophoresis	Separation by mass and charge, identification using specific antibodies	Long	High	Reference method [5,15,23]

Practical implications for laboratories

Three recommendations follow from the material compiled above and require no new studies. Each laboratory performing PEG precipitation should establish its own decision thresholds for the platform it uses [8]. Samples suspected of containing a macroenzyme should be tested alongside a control sample with similar activity of the enzyme in question, but without any suspicion of a macroenzyme [8,32]. The source of the threshold used should be clearly indicated because the same value is attributed to different sources or given without one [18,28,32].

Proposed management algorithm

Failure to recognize a macroenzyme may lead to costly or invasive diagnostic procedures. Galasso et al. noted that, in individual patients, liver scintigraphy or biopsy was performed during the diagnostic process before the diagnosis of macro-AST, and the diagnosis of a macroenzyme was recorded in the medical records in only nine of 42 patients [10]. Liver biopsies were also performed on the patients described by Sharma et al. and Pei et al.: in both studies, the macroenzyme was diagnosed only after biopsy [28,44]. Figure 5 illustrates the proposed algorithm for managing isolated, persistent hyperenzymemia.

**Figure 5:**
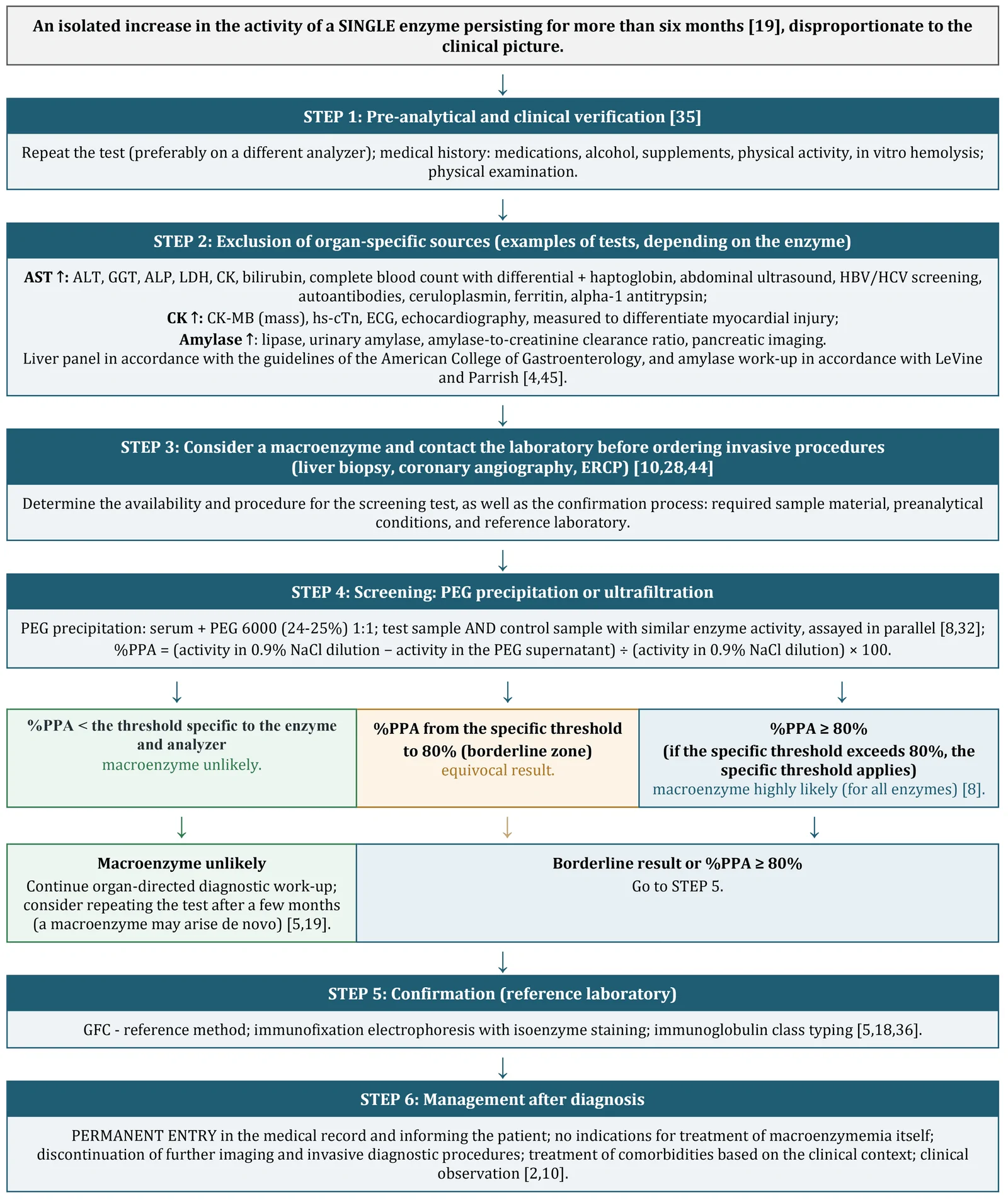
Proposed algorithm for managing isolated, persistent hyperenzymemia that is disproportionate to the clinical picture. Liver panel in accordance with the guidelines of the American College of Gastroenterology [4]; amylase workup in accordance with LeVine and Parrish [45]. Management after diagnosis in accordance with Remaley and Wilding [2], and Galasso et al. [10]. The three-zone rule in Step 4 combines the enzyme- and platform-specific thresholds compiled in Table 4 with the ≥80% rule proposed by Bürki et al. [8]; the intermediate zone is a construct of this review and has not been prospectively validated. Prepared by the authors based on data compiled in this review. PEG - polyethylene glycol; %PPA - percentage of activity precipitated by PEG; hs-cTn - high-sensitivity cardiac troponin; HBV/HCV - hepatitis B/C virus; ECG - electrocardiography; ERCP - endoscopic retrograde cholangiopancreatography; GFC - gel filtration chromatography; AST - aspartate aminotransferase; ALT - alanine aminotransferase; GGT - gamma-glutamyl transferase; ALP - alkaline phosphatase; LDH - lactate dehydrogenase; CK - creatine kinase; CK-MB - cardiac isoenzyme of creatine kinase; NaCl - sodium chloride

Areas for further research

The effects of the lack of harmonization of %PPA thresholds are most evident in the range of intermediate values. This can be seen both in cystic fibrosis patients with electrophoretically confirmed macro-ALT, whose diagnosis depended on the threshold used, and in the archived samples from Dedeene et al., which were negative at the commonly used 73% threshold despite confirmation by another method [21,25]. Multicenter studies are needed to establish decision thresholds for %PPA that are specific to the enzyme, analytical platform, and age, with validation of the thresholds against electrophoresis or GFC. The study by Čásenská et al. addresses the enzyme-, platform-, and age-specific part of this requirement but was conducted in a single center [32]. At the same time, it is necessary to standardize the protocol because without agreeing on the concentration and type of PEG, the temperature and incubation time, the centrifugal force, and the reference material, comparison of thresholds between centers remains unreliable.

The effect of pyridoxal phosphate activation requires separate investigation. Litin et al. demonstrated that in three patients with macro-AST, activation with pyridoxal phosphate increased the measured AST activity 2.4- to 12-fold compared with the assay performed without the cofactor [26]. They hypothesized that macro-AST began to be described only after the introduction of this cofactor into the reagents [26]. Fermon et al. observed the same effect on a current platform, with a ratio of activity measured with pyridoxal phosphate to activity measured without it of 27.8 in a patient with macro-AST against 1.10 in a control sample, and confirmed experimentally the observation of Litin et al. that the cofactor does not dissociate the complex [26,41]. The %PPA in that report was determined using the supplemented assay, and the polymer is reported as PEG-600, so the protocol is not included in Table 3. The effect of activation on the %PPA value itself needs to be verified, since %PPA is the ratio of two measurements performed using the same reagent. This may have practical significance because some of the thresholds were determined on samples without pyridoxal phosphate activation, and their transfer to a platform using this cofactor has not been verified [25].

The presence of a macroenzyme is not always a stable condition over time. A macroenzyme may persist for many years from childhood, disappear, or appear de novo in an adult. Caropreso et al. found that macro-AST persisted in six of nine children re-examined after an average of six years, while Aleknavičiūtė-Valienė et al. documented macro-AST first detected at the age of 71 [5,19]. The frequency of individual macroenzymes varies. In the cohort studied by Marczak-Karpina et al., macro-CK, macroamylase, and macro-GGT were the most common, while macro-ALT was not detected even once [20]. Findings for macro-AST should not be generalized to other enzymes. Čásenská et al. postulate the use of age-dependent decision limits for ALT, ALP, CK, and lipase [32].

Limitations

This study is a narrative review rather than a systematic one. The narrative form was chosen because the quantities compared here are not commensurable as summary estimates: thresholds determined on different analytical platforms, in different populations, and by different statistical rules would not pool into a value that could be referred back to any one platform or population, and no single appraisal tool grades case reports, reference-range studies, and retrospective reviews on a common scale. The review was not registered in advance; the search and the selection criteria are stated in this report so that the selection can be audited, but the review is not presented as compliant with the Preferred Reporting Items for Systematic Reviews and Meta-Analyses (PRISMA) statement and includes no formal assessment of the risk of bias. The proposed algorithm synthesizes practice described in the cited reports and has not been prospectively validated.

Several features of the search may have introduced selection bias. Two databases were searched, PubMed and the Polish Medical Bibliography, with no coverage of Embase, Scopus, or Web of Science, and no search of grey literature, preprints, or conference proceedings. The primary search used a single term. A broader set of terms run over the same period retrieved approximately eight times as many records, of which two met the inclusion criteria and were included, indicating that the primary term alone was not comprehensive. A large proportion of the additional yield concerned postoperative hyperamylasemia after pancreatic surgery and endoscopic retrograde cholangiopancreatography, a separate body of literature; the remaining records were screened at title and abstract level rather than in full text, and none were identified as contributing a value of a kind listed in the inclusion criteria that was not already represented.

Backward citation searching was limited to three citation steps, and forward citation searching covered only the four datasets that established the decision thresholds, so reports citing the other included studies may have been missed. The database searches covered publications from 16 March 2021 onwards, so systematic coverage applies to that period only, and the earlier literature, including two of the four datasets establishing %PPA thresholds, entered through the reference lists of the retrieved reports and, in one case, through forward citation searching. Publications were restricted to English, Polish, and French. Reports of positive findings are more likely to be published than negative ones, which is relevant to the detection rates compiled in Table 2.

All reports meeting the inclusion criteria are listed at study level in Table 1. Their allocation to the detailed comparison tables follows the scope stated for each of those tables, and further reports meeting the inclusion criteria may exist that were not identified by the search. Because a report qualified only if it contributed a value not already represented, this review maps the values in circulation rather than the literature as a whole, and the number of reports listed for a given threshold or protocol is not an estimate of how widely it is used. Three reports retrieved by the database searches met every other criterion and were excluded solely because the values they reported were already represented, as shown in Figure 1. The evidence behind any single value is therefore not visible in the tables: a threshold reported once and a threshold reported repeatedly appear alike, and no conclusion about how well a value is supported should be drawn from this review.

The numerical summaries presented in Tables 2, 4, 5 combine data obtained using different methods, at different thresholds, and in different populations. For this reason, these summaries should be treated as maps of discrepancies. The thresholds of Wyness et al. were included in the summary after converting the recovery rate to %PPA; due to the inverse metric and the two-sided nature of the reference interval, these values should be treated as approximate. The thresholds of Wyness et al. were also determined in comparison with a control group consisting of apparently healthy subjects and using PEG 8000 [22,36], while the other studies are based on samples from patients with elevated activity [8,23] or normal and elevated activity [25].

## Conclusions

PEG precipitation is one of the most accessible macroenzyme screening methods compared in this review. Yet its decision thresholds diverge widely. For the same enzyme, one number can support opposite conclusions depending on which limit a laboratory has adopted. A %PPA value should therefore be reported together with the threshold applied to it, the analytical platform used, and the control assayed alongside it. Where the value falls in relation to that threshold affects whether isolated hyperenzymemia is attributed to a macroenzyme or to organ damage, whether treatment is continued or withdrawn, and whether the patient undergoes further, sometimes invasive, investigation.

The same lack of standardization runs through the precipitation protocols themselves and through the cheaper alternative of storing the sample at 4°C, which did not reliably exclude a macroenzyme in the series compared here. A single sample may therefore be classified differently in two laboratories, and the source of that discrepancy is the procedure rather than the patient. Closing that gap will require enzyme-specific and platform-specific decision thresholds, established on defined reference populations, validated against a confirmatory method, and derived from an agreed precipitation protocol. Until that work is done, referral to a reference laboratory for confirmation remains necessary whenever the screening result is equivocal or clinically decisive.
